# Appropriateness of Antibiotic Prescriptions for Urinary Tract Infections

**DOI:** 10.5811/westjem.2020.1.45944

**Published:** 2020-04-13

**Authors:** Paige C. Chardavoyne, Kathryn E. Kasmire

**Affiliations:** * Pennsylvania State College of Medicine, Hershey, Pennsylvania; † Penn State Health Milton S. Hershey Medical Center, Division of Emergency Medicine, Hershey, Pennsylvania

## Abstract

**Introduction:**

Urinary tract infections (UTI) are a common indication for antibiotic use in the emergency department (ED). With antibiotic resistance on the rise, it is essential that antibiotics be prescribed appropriately for UTIs. Our objective was to evaluate the appropriateness of antibiotic prescriptions by ED providers for uncomplicated cystitis and pyelonephritis.

**Methods:**

We conducted a retrospective study of females ages 2–50 years seen in an academic ED from January 2017 to April 2018 diagnosed with UTI. We assessed the appropriateness of discharge antibiotic prescriptions, as determined by adherence to clinical practice guidelines, best evidence for the particular indication (cystitis vs pyelonephritis for children and adults), and the local antibiogram.

**Results:**

A total of 421 patients were included in this study. Of these, 60 children and 198 adults were diagnosed with cystitis, and 47 children and 116 adults were diagnosed with pyelonephritis. Treatment in the absence of true infection was common, with culture-confirmed UTI occurring in only 17/50 (34%) of children and 60/129 (47%) of adults diagnosed with cystitis, and 23/40 (58%) of children and 58/87 (67%) of adults diagnosed with pyelonephritis, among patients who had urine cultures. The type of antibiotic prescribed was appropriate in 53/60 (88%) of children and 135/198 (68%) of adults with cystitis, and 38/47 (81%) of children and 53/116 (46%) of adults with pyelonephritis. The most common inappropriate antibiotic types were beta-lactams in adults (n = 92), nitrofurantoin for pyelonephritis (n = 16), and amoxicillin (n = 15). Dosing and duration errors were also common, occurring in 122/279 (44%) of prescriptions of an appropriate antibiotic type. The frequency of errors in the type of antibiotic prescribed was similar among provider types (attending physician, resident physician, and advanced practice clinician; p = 0.926).

**Conclusion:**

This study reveals room for improvement in antibiotic prescription practices across provider cohorts in the ED for the management of uncomplicated cystitis and pyelonephritis in females.

## INTRODUCTION

Urinary tract infections (UTI) are common among females, accounting for upward of 10.5 million visits to physician offices and emergency departments (ED) per year.^1^ While it is important to promptly treat UTIs with antibiotics to prevent complications such as septic shock and renal scarring in children,^2^,^3^ it is also prudent to use antibiotics only when needed in an effort to reduce adverse medication effects and antibiotic resistance. With common UTI pathogens, resistance to fluoroquinolones and trimethoprim-sulfamethoxazole (TMP-SMX) has been on the rise. Additionally, there has been increasing prevalence of extended-spectrum β-lactamase and other multi-drug resistant organisms.^4^–^6^

Given the implications of inappropriate antibiotic prescription practices to individuals and populations, it is essential that providers appropriately select antibiotic treatment for the management of suspected UTIs. Guidelines from sources such as the Infectious Diseases Society of America (IDSA) and the American Academy of Pediatrics (AAP) can provide guidance.^7^,^8^ However, local factors, such as site-specific antibiotic resistance, also must be taken into account. Most organizations now publish hospital-wide antibiograms, which were present in 98% of United States hospitals in 2014.^9^ Although hospital antibiograms exist, it remains unknown whether providers are using this tool, in conjunction with guidelines, such as those from the IDSA, to tailor their prescription practices to their particular location. In a recent survey of pediatric providers, 70% reported access to local antibiograms and, of these, only 50% reported using the antibiogram “always” or “most of the time” when empirically prescribing antibiotics for UTIs.^10^ Prescribing practices for UTIs are variable and problems exist, including the use of overly broad antibiotics and treatment in the absence of true infection.^11^,^12^

To investigate antibiotic prescribing practices for suspected UTIs, we primarily sought to determine whether empiric antibiotic selection was appropriate for suspected UTIs in children and adults. Secondarily, we set out to analyze frequency of antibiotic prescription in the absence of true infection and whether variation in appropriate antibiotic selection existed between specific provider groups, including attending physicians, resident physicians, physician assistants, and nurse practitioners. We hypothesized that inappropriate antibiotics were frequently prescribed, that patients were frequently treated with antibiotics in the absence of true infection, and that variation existed between provider groups.

## METHODS

### Study Design

The study was a retrospective analysis of females ages 2 to 50 years diagnosed with a UTI who were treated and discharged between January 1, 2017 – April 16, 2018, from an academic medical center ED. Females age 18 years or older were categorized as adults, while females under the age of 18 years were categorized as children. Institutional review board approval was obtained. We identified patients via an ED diagnosis of one or more of the following *International Classification of Diseases* (ICD-10) codes for UTI: ICD-10 CM: N39.0 (UTI, site not specified); N30.00 (acute cystitis without hematuria); N30.91 (cystitis, unspecified with hematuria); N30.80 (other cystitis without hematuria); N30.0 (acute cystitis); N30.01 (acute cystitis with hematuria); N30.90 (cystitis, unspecified without hematuria); N30 (cystitis); N30.8 (other cystitis); N30.9 (cystitis, unspecified); N30.81 (other cystitis with hematuria); N10 (acute pyelonephritis); and N12 (pyelonephritis). All data collection was conducted by PC after receiving training from KK. Approximately 5% of charts were reviewed by both authors independently to assess for congruency, including random charts and any cases in which there was uncertainty.

We excluded patients from this study if they had UTI complications (including renal abscess, septic shock, concurrent nephrolithiasis, or required hospital admission or observation); had pre-existing renal or urologic disease (including vesicoureteral reflux or other functional abnormality; obstructive uropathy, permanent or intermittent catheter, history of urinary tract surgery); were pregnant or up to six-weeks post-partum; were prisoners; had diabetes mellitus; or were immunocompromised. This included patients taking medications such as steroids, biologics, and chemotherapeutic drugs. We also excluded patients with recurrent UTI (had a UTI with or without antibiotic treatment within the prior month) and patients who were already prescribed an antibiotic for the current UTI prior to the ED visit.

**Population Health Research Capsule**

What do we already know about this issue?

Previous studies have found overuse of antibiotics for urinary tract infections (UTI).

What was the research question?

To evaluate the appropriateness of antibiotic prescriptions for UTIs.

What was the major finding of the study?

Antibiotics were overused and inappropriate antibiotics were commonly prescribed for suspected UTIs.

How does this improve population health?

This study identified antibiotic misuse including overly broad antibiotics and overdiagnosis of UTIs, which can promote antimicrobial resistance.

Patients were categorized as having uncomplicated cystitis or pyelonephritis based on their ICD-10 code. In cases where the ICD-10 code CM N39.0 for “urinary tract infection, site not specified” was used, the authors categorized patients as having either cystitis or pyelonephritis based on medical chart documentation of symptoms, vital signs, physical exam, and provider impression. Patients were assigned to the pyelonephritis cohort if they had fever, flank pain, or costovertebral angle tenderness.^13^

### Outcomes and Data Analysis

The primary outcome was the appropriateness of the antibiotic prescription for UTI, including the antibiotic type, dose, and duration. We determined a list of appropriate discharge antibiotics (Table 1, Appendix Tables 1 and 2) using guidelines from the IDSA, the AAP, UpToDate, and our hospital’s 2016 outpatient antibiogram (Appendix Figure 1).^7^,^8^,^13^–^15^ These references were selected as they represent current and reputable sources used by providers in our ED. For children, there are many potential antibiotic options with limited evidence for superiority of a particular antibiotic or regimen.^16^ Thus, any antibioitcs recommended by expert sources^7^,^14^,^15^ with favorable susceptibility at our institution (> 80% of *E. coli* isolates susceptible) were included. For adults, antibiotics recommended by IDSA guidelines were used.^8^ Based on our local susceptibilities (Appendix Figure 1), all of the antibiotic options had favorable resistance patterns to *E. coli* (more than 80% of isolates susceptible) and were included as appropriate.

We did not include beta-lactams as appropriate options for adults in accordance with the IDSA guidelines, which recommend against routine use for UTI due to inferior efficacy, as there are numerous other antibiotic options for adults with favorable local susceptibilities. Allergies were reviewed during chart review, and no patients were allergic to all appropriate first-line options. There are no standardardized antibiotic recommendations in place in our ED. Our hospital antibiogram is available on the hospital infonet, and pocket cards are distributed periodically. It provides susceptibilities for various organisms for inpatients and outpatients, but does not provide empiric treatment recommendations for infection types, such as UTI.

The secondary outcomes were the frequency of positive urine culture (when obtained) and the appropriateness of discharge antibiotic prescriptions between provider types (resident physician, advanced practice clinician, and attending physician). UTI was defined as growth of greater than or equal to 100,000 colonies of a single uropathogen via clean catch, growth of greater than or equal to 50,000 colonies of a single uropathogen via direct catheterization, or growth of greater than or equal to 50,000 colonies of a single uropathogen via clean catch in the presence of a positive urinalysis.^13^,^14^ We performed data analysis using descriptive statistics and chi-squared tests where applicable.

## RESULTS

**Figure 1:**
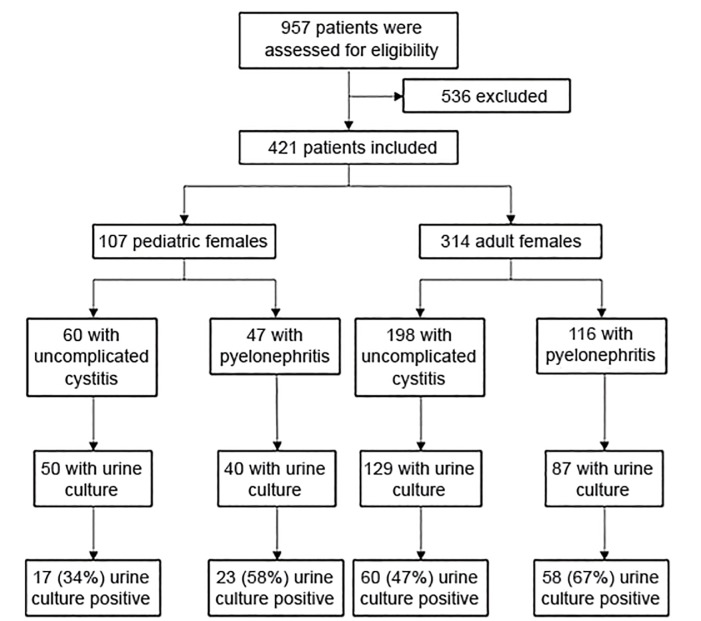
Flow diagram of study participants diagnosed with urinary tract infections.

During the study period, 421 patients met inclusion criteria (Figure 1). Common reasons for exclusion in adults included diabetes mellitus, presence of nephrolithiasis, and current treatment with antibiotics or immunosuppressing medications. Common reasons for exclusion in children included vesicoureteral reflux and current antibiotic treatment. Of the total 421 patients, 258 (61.3%) were classified as having cystitis and 163 were classified as having pyelonephritis (38.7%) (Figure 1). The median age for children was nine years and the median age for adults was 27 years.

Urine cultures were performed in 73% of patients (306/421), with higher utilization of urine culture in patients with pyelonephritis (127/163, 78%) than in patients with cystitis (179/258, 69%) (Figure 1). The presence of UTI was not confirmed for a substantial proportion of patients diagnosed with cystitis, whereas more patients diagnosed with pyelonephritis had a culture-proven UTI (Figure 1). A vast majority of the true positive urine cultures grew *E. coli* (80%).

**Figure 2:**
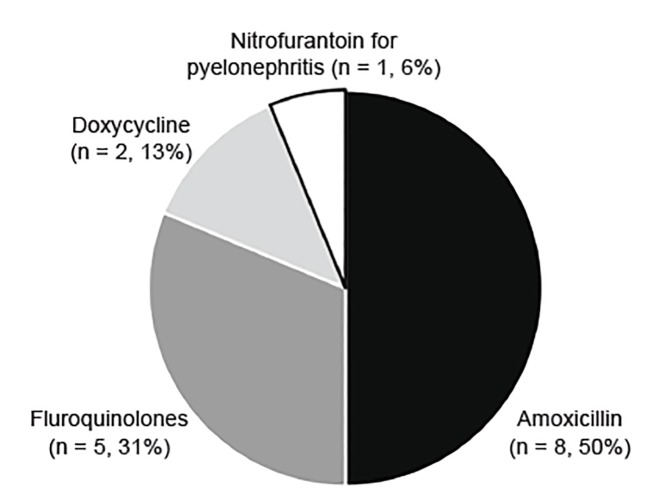
Inappropriate antibiotic types prescribed for children with urinary tract infections.

**Figure 3:**
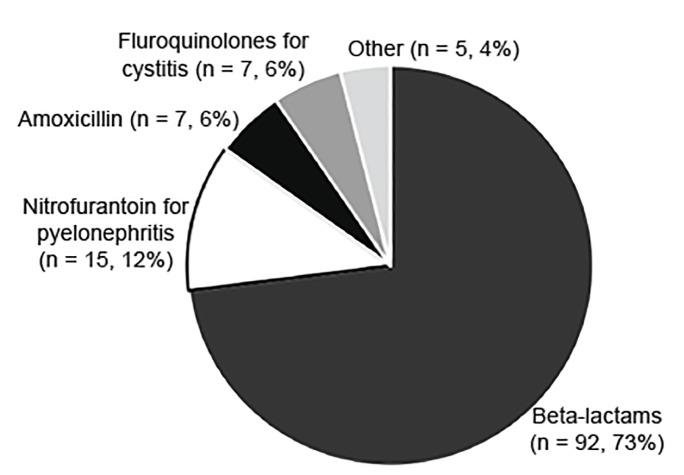
Inappropriate antibiotic types prescribed for adult women with urinary tract infections.

The appropriateness of discharge antibiotic prescriptions (Appendix Figure 2) is shown in Table 2. The majority of patients received an appropriate antibiotic type, except for adults with pyelonephritis (46%). Figures 2 and 3 detail the reasons for lack of accordance with clinical guidelines for antibiotic type in children and adults, respectively. Errors in dosing and duration were common, with only 157 (56%) of those with appropriate antibiotic type having correct dosing and duration when compared to our established criteria (Appendix Tables 1 and 2). Duration was more frequently incorrect than dosing. Antibiotics were commonly prescribed for a longer, rather than shorter, duration than is specified in the Appendix Tables 1 and 2. Of the 104 patients with incorrect duration, 73 patients (70%) received antibiotics for a longer duration and 31 patients (30%) received antibiotics for a shorter duration.

A total of 130 unique prescribers treated the 421 patients included in the study. We assessed the frequency of appropriate discharge medication prescription type, independent of dosage and duration, for cystitis and pyelonephritis combined across the following three cohorts: attending physician (18/27, 67%); resident physician (168/249, 67%); and advanced practice clinician (93/145, 64%). No difference was found between the groups (p = 0.926).

## DISCUSSION

In this study we found a number of areas for improvement in the initial management of female UTIs in an academic ED. Furthermore, we demonstrated that opportunity for improvement exists across all provider cohorts studied. These findings can guide improvement efforts for judicious use of antibiotics. Overtreatment of UTI was common, in particular for cystitis. As many as two-thirds of children who were treated for cystitis had a negative urine culture. Strategies to improve diagnostic accuracy and/or decrease unnecessary antibiotic treatment include the use of a decision aid, such as that by McIsaac et al for females with suspected cystitis.^17^ This simple decision aid using three variables (dysuria, leukocytes greater than a trace amount, and positive nitrites) was statistically associated with a positive urine culture result.

Strategies to reduce antibiotic overuse include delaying antibiotic prescription if infection is uncertain and discontinuing antibiotics if the urine culture results as negative. In a study by Knottnerus et al, many women were willing to delay antibiotic initiation for cystitis while awaiting urine culture result. More than half of these women experienced resolution of symptoms within a week without antibiotic therapy.^18^ In the pediatric and adult settings, nurse, clinician, and pharmacist follow-up after urine culture result has led to decreased total antibiotic days.^19^,^20^ A common theme is the need for improved, ED-based antimicrobial stewardship strategies, as the ED is unique and may require its own specific interventions. 21

Overtreatment of UTIs was also found in the form of excessive antibiotic duration (Table 2), particularly for cystitis. The variation in length of antibiotic courses could be due in part to limited evidence for superiority of particular regimens, especially for children.^16^,^22^ However, longer than recommended antibiotic courses can contribute to antibiotic resistance.^23^ Potential strategies for improving dosing and duration include interventions to the electronic health record, which have been demonstrated to improve appropriate antibiotic delivery.^24^,^25^

Finally, we investigated the utilization of appropriate antibiotic types given the local antibiogram and evidence-based guidelines. Adherence to the IDSA recommendations can increase narrow spectrum antibiotic use and decrease unnecessary antibiotic days.^26^,^27^ In our study, we found overuse of fluoroquinolones for cystitis (albeit at lower rates than in other reported studies).^26^,^27^ This antibiotic class can cause serious advserse events, promote antibiotic resistance, and predispose to *Clostridum difficile* infections.^28^–^31^ Other deviations from the guidelines included the use of potentially ineffective antimicrobials, such as amoxicillin and nitrofurantoin for pyelonephritis. While clinical outcomes were not tracked, the liklihood of treatment failure is high with these agents, with *E. coli* resistance to amoxicillin being widespread.^8^

The most common deviation from IDSA guidelines in adults was treatment with beta-lactams (Figure 3). The significance of this is unclear despite how common this practice is at our institution. These antibiotics are likely prescribed because local susceptibilities are favorable for 1^st^- and 2^nd^-generation cephalosporins. Furthermore, additional studies have been published since the IDSA guidelines in 2011 supporting efficacy of beta-lactams for the treatment of UTIs in adults.^32^–^35^ The use of beta-lactams could be studied further to provide more potential treatment options, especially given that antibiotic resistance to many other antibiotic classes is on the rise.

## LIMITATIONS

This study analyzed adherence to clinical guidelines and expert recommendations, assuming that these promote optimal care. Additional limitations of this study are associated with its retrospective design. The determination of antibiotic appropriateness relied on diagnosis of either cystitis or pyelonephritis; however, some patients had a non-specific diagnosis of “urinary tract infection, site not specified.” In this case, they were assigned to either the cystitis or pyelonephritis group based on the study team’s interpretation of the patient’s signs and symptoms. Errors in this designation by the study team would have affected the assessment of the appropriateness of antibiotics received. In an effort to check cohort assignment, we performed statistical analysis and found no significant difference in the appropriateness of discharge antibiotic type between patients whom the study team assigned to cystitis vs pyelonephritis and patients who had an ED diagnosis code for cystitis or pyelonephritis specifically (p > 0.05). Additionally, we did not assess why providers chose antibiotics that deviated from the recommendations, such as resistance in past urine cultures or lack of awareness of the guidelines. Furthermore, urine culture was used to assess presence of true infection; however, not all patients received cultures, which could affect our conclusions about the prevalence of true UTI in our study.

## CONCLUSION

This study reveals gaps in the management of children and adults with urinary tract infections, including administration of antibiotics in the absence of true infection and inappropriate choice of discharge antibiotic type, dose, or duration. These shortcomings occurred across all three provider types studied and represent areas for further education and quality improvement to promote antimicrobial stewardship.

## Supplementary Information



## Figures and Tables

**Table 1:** List of appropriate antibiotic types for the management of uncomplicated cystitis and pyelonephritis.

Age group	Infection type	Appropriate medications
Children^14^,^15^	Uncomplicated cystitis	Amoxicillin / clavulanic acid (immediate release formulations) 1st–3rd generation cephalosporin Nitrofurantoin Trimethoprim-sulfamethoxazole
	Pyelonephritis	Amoxicillin / clavulanic acid (immediate release formulations) 1st–3rd generation cephalosporin Trimethoprim-sulfamethoxazole
Adults^8^,^13^	Uncomplicated cystitis	Fosfomycin Nitrofurantoin Trimethoprim-sulfamethoxazole
	Pyelonephritis	Ciprofloxacin Levofloxacin Trimethoprim-sulfamethoxazole

**Table 2:** Appropriateness of antibiotic prescriptions for urinary tract infection.

Age group	Children, n (%)		Adult, n (%)	
Diagnosis	Cystitis	Pyelonephritis	Cystitis	Pyelonephritis
Total	60 (14%)	47 (11%)	198 (47%)	116 (28%)
Appropriate discharge antibiotic type	53/60 (88%)	38/47 (81%)	135/198 (68%)	53/116 (46%)
+ Appropriate dose^a^	48/60 (80%)	36/47 (77%)	128/198 (65%)	49/116 (42%)
+ Appropriate dose and duration^b^	41/60 (68%)	22/47 (47%)	68/198 (34%)	26/116 (22%)

a Appropriate antibiotic type and correct dose.

b Appropriate antibiotic type and correct dose and duration.
