# Supplementary material for: Appropriateness of Antibiotic Prescriptions for Urinary Tract Infections
Source: West J Emerg Med. 2020 Apr 13;21(3):633–9. doi: 10.5811/westjem.2020.1.45944 (PMC7234695; doi:10.5811/westjem.2020.1.45944)
Supplement: Supplementary file 1 [file wjem-21-633-s001.docx]

**Appendix**

**Table 1.** Duration and dosing of appropriate oral antibiotics for the management of cystitis and pyelonephritis in pediatric females.^14 †^

| **Antibiotic** | **Dose^*^** | **Max dose** |
| --- | --- | --- |
| Amoxicillin-clavulanic acid | 22.5 mg amoxicillin/kg BID  or 20-40 mg amoxicillin/kg/day TID | 875 mg amoxicillin |
| Cephalexin | 25-50 mg/kg/day BID to QID | 500mg |
| Cefdinir | 14 mg/kg/day once daily or BID | 600 mg |
| Cefixime | 4 mg/kg BID | 200 mg |
| Cefpodoxime | 5 mg/kg BID | 200 mg |
| Cefprozil | 15 mg/kg BID | 500 mg |
| Ceftibuten | 9 mg/kg once daily | 400 mg |
| Cefuroxime | 15 mg/kg BID | 500 mg |
| Nitrofurantoin | 5-7 mg/kg/day BID to QID | 100 mg |
| Trimethoprim-sulfamethoxazole | 4 to 6 mg trimethoprim/kg BID | 160 mg trimethoprim |

^*^A dose within 10% of our guidelines was considered appropriate medication dosing

^†^ Antibiotic duration of 2-7 days for cystitis and 10-14 days for pyelonephritis was considered appropriate^23,26^

**Table 2.** Dosing and duration of appropriate oral antibiotics for the management of cystitis and pyelonephritis in women.^8,13^

| **Antibiotic** | **Dose** | **Duration** |
| --- | --- | --- |
| Ciprofloxacin | 500mg BID, or 1000mg XR daily | Pyelonephritis: 5-7 days |
| Fosfomycin | 3g | 1 dose |
| Levofloxacin | 750mg daily | Pyelonephritis: 5 days |
| Nitrofurantoin | 100 mg BID | Cystitis: 5 days |
| Trimethoprim-sulfamethoxazole | 160/800mg BID | Cystitis: 3 days  Pyelonephritis: 14 days |


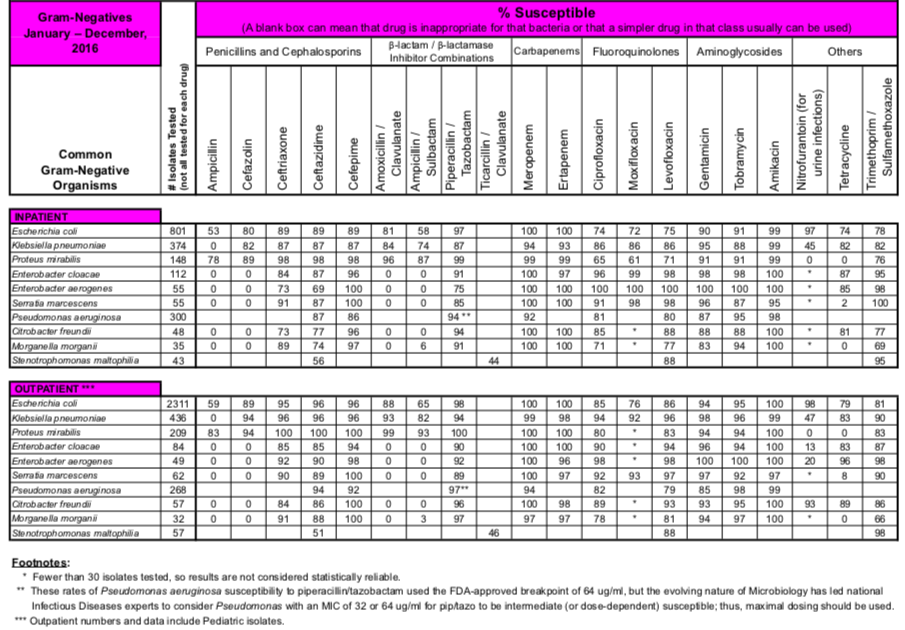


**Figure 1.** Hospital inpatient and outpatient antibiogram. The outpatient antibiogram includes Emergency Department patients and was used in this study.





**Figure 2.** Antibiotics prescribed for uncomplicated cystitis and pyelonephritis. *TMP-SMX= trimethoprim-sulfamethoxazole.*
